# Significant others in inflammatory arthritis: roles, influences, and challenges—a scoping review

**DOI:** 10.1007/s00296-024-05639-9

**Published:** 2024-07-06

**Authors:** Charlotte Werdal Hansen, Marianne Wetendorff Nørgaard, Annette de Thurah, Julie Midtgaard, Pernille Fevejle Cromhout, Bente Appel Esbensen

**Affiliations:** 1 Department for Rheumatology and Spine Diseases, Center for Arthritis Research (COPECARE), Centre of Head and Orthopedics, Righospitalet, Glostrup, Denmark; 2 Danish Centre of Systematic Reviews: A JBI Centre of Excellence, Center for Clinical Guidelines, Faculty of Medicine, Aalborg University, Aalborg, Denmark; 3 Department of Rheumatology, Aarhus University Hospital, Aarhus, Denmark; 4 Department of Clinical Medicine, Aarhus University, Aarhus, Denmark; 5 Mental Health Center Glostrup, CARMEN (Centre for Applied Research in Mental Health Care), Copenhagen University Hospital-Mental Health Services CPH, Copenhagen, Denmark; 6 Department of Clinical Medicine, Faculty of Health and Medical Sciences, University of Copenhagen, Copenhagen, Denmark; 7 Novo Nordisk A/S, Søborg, Denmark

**Keywords:** Rheumatoid arthritis, Spondylo arthritis, Psoriatic arthritis, Disease management, Patient education, Family nursing

## Abstract

Improving self-management in individuals with inflammatory arthritis (IA) is crucial for effective disease management. However, current recommendations primarily focus on interventions for the diagnosed individuals, overlooking the potential impact of their significant others on their self-management abilities. This review aims to fill this gap by identifying and mapping relevant research employing both qualitative and quantitative design to provide a broader understanding of the potential of significant others in relation to IA management. We examined studies published from 2007 to 2024 that explore our research questions using electronic databases and grey literature searches. Two independent reviewers meticulously screened and categorized the studies based on a developed framework employing basic content analysis. Out of 20.925 studies, 43 were included: 22 quantitative studies (including 1 educational trial), 20 qualitative studies, and 1 mixed-methods study. Our analysis of the included studies revealed that significant others predominantly provided practical and emotional support and could positively or negatively influence the person with IAs self-management abilities. Additionally, significant others reported their own feelings of emotional distress and expressed the need for knowledge, skills and social support enabling them to provide better support while taking care of them self. Greater focus on the significant others of those diagnosed with IA in their provision of support to this patient group may both improve the people with IA self-management skills and address significant others’ reported needs. Future studies should explore the impact of such initiatives through randomized controlled trials.

**Supplementary Information:**

The online version contains supplementary material available at 10.1007/s00296-024-05639-9.

## Introduction

Inflammatory arthritis (IA) encompasses various progressive and fluctuating autoimmune diseases causing unpredictable pain, morning stiffness, impaired physical functioning, fatigue, depression, anxiety, and diminished quality of life [1, 2]. Contemporary IA treatment typically involves pharmacological treatment and disease monitoring through outpatient visits and minimal support from health professionals within rheumatology (HPR). Subsequent treatment recommendations include focus on improving the self-management skills of individuals diagnosed with IA through interventions directed solely at the patients themselves [3–5].

From a family-system theory approach, disease management is however, not limited to the internal abilities of the person diagnosed but is heavily influences by the individuals who surround them through their emotional interconnectedness [6]. Different terminologies such as caregiver, family, support system etc. are applied within the family-system theory and research in general to describe the individuals surrounding the person diagnosed. We prefer the terminology significant others, which according to the Merriam Webster definition includes any individual important to a person overall well-being, and therefore often includes close family relations but do not exclude non-family members [7, 8]. Furthermore, the family-system approach recognizes that diseased do not only affect the person diagnosed, but also the lives of the significant others, and that health professionals must also understand the challenges and needs of the significant others and the emotional interconnectedness between the person diagnosed and the significant other, to improve how both approach disease management [6].

However, research on what supportive task significant others to people with IA have, whether the significant others influence the individuals with IA self-management abilities and what challenges and needs the significant others experience in relation to delivering support, is easily identified and accessed. Preliminary searches in PubMed, the Joanna Briggs Institute (JBI) Database of Systematic Reviews, and the Cochrane Database of Systematic Reviews indicate that some research exists on the support, influence, challenges and needs of significant others concerning IA self-management, but a comprehensive overview is lacking. Thus, *this scoping review aims to fill this gap by identifying and mapping relevant research employing both qualitative and quantitative designs to provide a broader understanding of the potential of significant others in relation to IA management.*

To guide the scoping review, we developed the following four research questions (RQ):

- RQ1. What has been reported on what the role of significant others to people with IA entail, from the respective perspectives of the patient, the significant other, and HPR?
- RQ2. What has been reported on significant others influence on people with IAs self-management abilities?
- RQ3. What has been reported on the challenges and resultant needs of these significant others?
- RQ4. What are the research gaps in the literature regarding significant others to people with IA?

## Materials and methods

This scoping review adhered to the JBI methodology for scoping reviews [9] and followed the reporting guidelines outlined in the Preferred Reporting Items for Systematic reviews and Meta-Analyses extension for Scoping Reviews (PRISMA-ScR) [10]. Additionally, a pre-review protocol was developed and registered in the Open Science Framework (OSF) registry (10.17605/OSF.IO/NRJCX) prior to commencing the review.

### Eligibility criteria

We included studies involving adults (≥ 18 years old) diagnosed with IA, their adult significant others (≥ 18 years old), or HPRs capable of addressing one or more of our four RQs. By including both studies applying a qualitative and quantitative design, it enabled us to understand our four RQ from different methodological perspectives. Specifically, we reviewed qualitative studies reporting on people with IA, their significant others, or HPRs view on what the role of significant others entailed, how significant others influences people with IAs ability to manage IA, and the challenges and needs of significant others. We also reviewed quantitative studies investigating support provided by significant others in relation to people with IAs symptom severity, disease severity, physical functioning, treatment adherence, and other patient-related outcomes influenced by self-management ability. We did not include non-systematic reviews or systematic reviews (this was to avoid including the same study twice, but we did check these to ensure we did not miss any studies). Protocols, expert opinions, or validation studies vas also excluded.

IA was limited to rheumatoid arthritis (RA), psoriatic arthritis (PsA), and spondyloarthritis (SpA) as per the European Alliance of Associations for Rheumatology (EULAR) definition [4]. Significant others were broadly defined as individuals of possible great importance to the person with IA based on the Merriam Webster definition [7] and included partners, adult children, parents, next of kin, informal caregivers, friends, and members of the patient’s social network. Given the notable advancements in the pharmacological treatment of IA in the last 15–20 years, our interest was specifically in the contemporary role, influence, challenges and needs of significant others supporting people with IA. Therefore, only studies published within the last 15 years (from 2007 onwards) were included in the review.

### Search strategy

Initial searches in PubMed (MEDLINE) were carried out to identify relevant key and index words, forming the basis for a comprehensive search strategy developed in collaboration with a research librarian and the research group (see Supplementary File 1). The study language was confined to English, Danish, Swedish, Icelandic, and Norwegian due to limitations in translation resources. Additionally, the search was constrained to studies published within the last 15 years (from 2007 onwards) as stated previously.

The search was executed between February 21st, 2023, and March 7th, 2023, as outlined in Supplementary File 2, and the search was updated again in April 2024. The database search encompassed MEDLINE (PubMed), Embase (Ovid), CINAHL (EBSCO), PsycInfo, Scopus and Cochrane Reviews. Unpublished studies were sought through Google Scholar and specific registries, including the CENTRAL register, OSF register, and PROSPERO register. Conference abstracts from the EULAR and the American College of Rheumatology (ACR) were also scrutinized. Furthermore, the identified reviews and the reference lists of included studies were examined to identify additional studies.

### Study selection and data extraction

Two reviewers (CWH and BAE) independently screened hits, first evaluating titles and abstracts and subsequently assessing full texts at Covidence.org, adhering to the predetermined eligibility criteria [9]. Exclusion reasons were documented, with disagreements resolved through discussion. The exclusion criterion “studies solely reporting one relevant sentence” was added during the process. Data extraction of the included studies (year, country, design, methodology, outcomes) was done by the reviewers using a extraction tool developed with the protocol (see Supplementary File 3). The extraction tool was piloted on three reports and adjusted to include information on patient–significant other relationships, study aims, and conclusions.

### Data analysis

Following the JBI methodology [11], we applied general basic statistics and narrative reporting to analyze and report on the characteristics of the included studies. In line with the JBI recommendations, scoping reviews aiming to identify concepts should utilize a framework for sorting and reporting findings from any source, including quantitative study reporting. However, as there is no established framework for categorizing and reporting extracted characteristics related to the role, challenges and needs of significant others in the included studies, JBI suggests applying basic qualitative content analysis to develop such a framework. Accordingly, we applied the four phases of Elo and Kyngäs’ qualitative inductive content analysis process on a manifest level to create a categorization framework [12]. (I) Two reviewers (CWH and BAE) familiarized themselves with the sources during the screening process; (II) CWH applied open coding by labeling the data sources in accordance with which RQs they answered (see Supplementary File 4); (III) an initial framework was developed by CWH and BAE to serve as a categorization tool; (IV) and all extracted data sources were sorted into the framework by CWH. Following the JBI methodology, a critical appraisal of the included studies was not performed. The final framework is available in Supplementary File 5.

## Results

### Search and screening

**Fig. 1:**
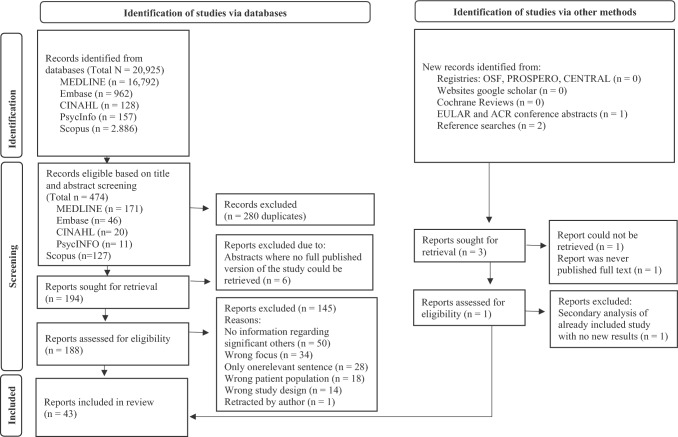
PRISMA flowchart

Out of the initial 20.925 hits, 188 records remained eligible for full-text screening after eliminating duplicates and those with unavailable full text. Ultimately, 45 records met the inclusion criteria by the conclusion of the screening process. Figure 1 provides specifics on the reasons for excluding full texts.

### Characteristics of included studies

Among the 43 included studies, 22 employed a quantitative design [13–34], 20 employed a qualitative design [8, 35–53], and 1 employed a mixed-methods design [54]. Notably, ten studies focused on investigating the role, challenges and needs of significant others [37, 39–41, 44, 47, 49, 50, 54, 55]. Seven studies explored characteristics or the prevalence of different characteristics within the significant others [13, 19, 25, 28, 30, 31, 51]. Sixteen studies examined the associations between support provided by significant others and various patient-related outcomes [14–18, 20–24, 26, 27, 29, 32–34]. Nine studies reported on the role of significant others within specific contextual situations, such as early referral decisions and strategies supporting medication use [35, 38, 42, 43, 45, 46, 51–53]. One study investigated the effect of an educational intervention targeting significant others [25]. For a detailed overview of the included studies, see Supplementary File 6.

### Characteristics of included participants

Participants in the included studies were mainly people with IA (*n* = 24 studies), followed by people with IA and their significant others (*n* = 14 studies). Five studies exclusively focused on significant others, and one study included rheumatologists as HPRs. Among the people with IA, RA was the predominant diagnosis (77%), with most being female (67%) and of a mean age of 53.4 years (SD 10.9, age range 18–92 years). When reported, significant others were most commonly the partners of people with IA (see Table 1).

**Table 1:** Characteristics of participants from the included studies included participants

	Study design			Total
	Quantitative	Qualitative	Mixed Methods	
**Total number of people (n)**	3880	1103	392	5375
IA diagnosis				
RA	3649	211	392	4,252 (79%)
AS	144	8		152 (3%)
SpA	14	36		50 (1%)
PsA	13	41		43 (< 1%)
Other IA diagnoses^a^		8		8 (< 1%)
Non-specified IA		799		799 (14%)
Non-IA^b^	60			60 (1%)
Age range in years	29–92	18–90		18–92
Age mean and (SD) in years calculated as mean reportings	53.8 (9.46)^c^	54.5 (10.2)^d^	51.8 (13.1)	53.4 (10.9)
Females; n (%)	2,798 (71%)	783 (70.9%)^e^	227 (58%)	67%
**Total number of significant others (n)**	1212	106	405	1723
Specified relationship between person with IA and significant other				﻿707 (41%)
Partners	427	68		495 (29%)
Parents	107	3		110 (6%)
Adult children ≥ 18 years	92	4		96 (5%)
Friends		6		6 (< 1%)
Non-specified relationship between person with IA and significant others				1016 (59%)
Non-specified family members	487	7		494 (29%)
Non-specified informal caregivers	38	18	405	461 (27%)
Not reported	61			61 (4%)
Age range in years	28–86	18–90		18–90
Age mean and (SD) in years calculated as mean reportings	53.5 (12.1)^f^	Not reported	Not reported	53
Female; n (%)	533 (44%)	50 (47%)^g^	190 (47%)	773 (45%)
**Total number of rheumatologists (n)**			141	141
Female; n (%)			42 (30%)	
Years in practice mean and (SD)			13.7 (5)	

*RA* rheumatoid arthritis, *AS* ankylosing spondylitis, *SpA* spondylo arthritis, *PsA* psoriatic arthritis

^a^Such as systemic lupus erythematosus, juvenile arthritis, and reactive arthritis

^b^One study, in addition to IA, also included people with osteoarthritis

^c^Mean and SD were reported in 85% of reports

^d^Mean and SD were reported in 11% of reports

^e^Sex was reported in 94% of reports

^f^Mean and SD were reported in 91% of reports

^g^Sex was reported in 89% of reports

### RQ 1. The role of significant others

17 studies applying a qualitative design, 3 studies applying a quantitative design, and 1 mixed-methods study reported findings regarding the role of significant others. All described the role of significant others as mostly providing practical and emotional support to the person with IA, with few studies reporting significant others providing more than support. Below, the specific findings from the included studies are reported based on categorization by the develop framework described in the methodology section.

**Table:** 

Practical support	***Activities of daily living****:* Helping with chores, household activities, getting dressed, etc. [35–37, 39, 40, 48–50] ***Medical care:*** Booking doctor appointments, driving the person with IA to consultations, picking up medication, administering and overseeing medication intake, seeking information, recognizing the need for a change in medication or assessing the IA patient’s reporting of their symptoms [37–40, 43, 46, 51, 54]. Two studies reported that 75% of significant others provide practical support with medical care [29, 54] ***Financial aid*:** Paying for medication or sharing accommodation as financial aid [39, 54]
Emotional support	***Helping patients deal with feelings including depression and anxiety*:** Motivating the person with IA to get up in the morning, go for a walk, and continue everyday life [35, 40, 50, 52] ***Medical care****:* Motivating the person with IA to seek medical help and adhere to treatment [39, 46] ***Most important:*** One study reported emotional support as the most important type of support delivered as it would persist even when formal caregivers were involved in the caretaking [50]
More than support	***Taking on a motherly role:*** Providing childcare and creating close relationships with the children of mothers with IA when the mother was incapable due to their IA [39, 42] ***(Over)protecting***: Ensuring the person with IA did not experience mood dips or disease flare-ups, although this was impossible[8, 44]

### RQ 2. Significant others’ influence on people with IA

17 studies applying a quantitative design, 123 studies applying a qualitative design, and 1 mixed-methods study reported on the significant others’ influence on the person with IA. This influence was reported to both positively and negatively influence the self-management abilities of people with IA, including their disease-, emotional-, and role management. The studies’ reported influences of significant others on these different areas of self-management as listed below.

**Table:** 

Disease management	***Disease activity****:* One study found that educating significant others in IA could decrease IA disease activity [25]. Another found that depression within significant others predicted higher reports of IA disease activity [24] ***Pain:*** Several studies reported on a relationship between perceived support by the person with IA and the level of pain they experienced [14, 15, 20, 23, 52]. Satisfaction with significant others’ support was reported to disrupt the negative effect of catastrophizing on pain [14]. Positive emotional support from significant others was reported to be associated with a decrease of pain in people with IA throughout the day, while negative support was reported to be associated with an increase of pain in people with IA throughout the day [15] ***Disability:*** Positive interactive relationship was found to inverse correlate with physical disability in people with IA [22]. A possible association between a lack of education in IA among significant others and an increase in patient disability was reported [25]. Having a depressed significant other was found to predict higher reports of disability in people with IA [24] ***Treatment adherence:*** Several studies reported that people with IA, significant others, and HPR believed that significant others provided valuable observations and reporting of the person with IAs condition and treatment adherence and that people with IA who received support from significant others were considered more likely to adhere to treatment [17, 32, 53, 54] ***Help-seeking behavior:*** Two studies reported that significant others could promote/lessen the IA patient’s help-seeking behavior by encouraging medical counseling to ensure diagnosis/treatment or suggesting alternative therapy instead of conventional therapy [38, 46] ***Self-efficacy:*** Good communication within the partnership, spouse satisfaction with social support, and low spousal burden were found to be associated with better self-efficacy in people with IA in one study [27]
Emotional management	***Anxiety, depression, and stress:*** Several studies reported an inverse association between support from significant others and IA patients’ symptoms of anxiety, depression, and stress [8, 13, 14, 20–24, 27, 34, 56]*.* One study found that the education of significant others may reduce symptoms of depression and anxiety in people with IA [25]. A better overall positive interactive relationship between people with IA and their significant others was also reported to predict fewer depressive symptoms [14]. One study reported that receiving support may reduce IA patients’ perceived level of stress [23] ***Quality of life:*** Support from significant others was reported to be associated with a higher quality of life in people with IA in one study [26]. Another study reported that problematic support affected the IA patient’s emotional well-being negatively [21]
Role management	***Identity and life roles:*** Significant others being overprotective, delivering help without being asked, and viewing people with IA as disabled or pitiful were reported to lead to feelings of unworthiness, humiliation, and being a burden, thereby having a detrimental impact on the person with IAs identity. However, when people with IA received support from significant others in a manner that met their needs and preferences and when the person with IAs identity and dignity were maintained, the reports found that the people with IA felt less like a burden and more confident [8, 36–38, 40–42, 44, 46, 49, 51, 53] ***Acceptance:*** When significant others accepted having IA, it was reported to be easier for the person with IA to accept it [41, 42, 52] ***Sickness-related absence:*** A higher degree of perceived support from significant others was reported to be associated with increased odds for sickness-related absence from work in people with IA [16]

### RQ 3. Challenges of significant others

10 studies applying a qualitative design and 6 studies applying a quantitative design reported primarily negative experiences or impacts of being a significant other of a person with IA and cited various needs of significant others related to their ability to cope with the burden. Secondly 2 studies applying a qualitative design and 2 studies applying a quantitative design reported on the importance of communication abilities in the relationship between the person with IA and their significant other, and the difference perspectives on how IA influenced the relationship between the person with IA and the significant other These are listed in the following.

**Table:** 

Life revolving around the person with IA	***Loss of social network:*** Significant others in the studies described experiencing isolation and a loss of their social network [37, 48, 55] ***Part of family life:*** The influence of IA was described as life-changing for the significant others, with all activities planned around the person with IAs ability to participate [8, 35, 36, 39, 44]. In one study, significant others described that, over time, adjusting their lives around the person with IA became a natural part of family life and increasingly took less effort [50]
Emotional and psychological impact	***Initial emotional reactions:*** Studies reported various negative emotions experienced by significant others, such as shock, frustration, sadness, and helplessness, upon the initial diagnosis ***Emotions related to IA pain:*** Feelings of distress and sadness were reported by significant others when watching the person with IA endure pain, alongside shifts in their mood. Additionally, frustration arose when the significant other was unable to alleviate the IA patient’s suffering ***Emotional overload:*** Studies reported that watching someone suffer from IA and delivering support could result in emotional overload and psychological distress [8, 37, 39, 44, 46, 48]. One study reported how significant others experienced emotional overload when the demands of the person with IA surpassed what they could provide or when they lacked sufficient time for self-care. This situation led to significant others neglecting their own needs, causing sadness, bitterness, and mood changes; the study defined this as emotional overload [48] ***Depression and stress:*** Being the significant other of an IA patient was associated with increased odds for experiencing symptoms of stress and depression [19, 28, 30, 31]. A study reported that 26.7% of significant others felt depressed and 80% burdened [28]. Burden was reported as the main stressor and interacted with patient disability. Significant others’ symptoms of depression were also reported to be positively associated with IA pain [19, 28, 30, 31]. One study reported that low levels of self-efficacy, mental health, or physical functioning in people with IA corresponded to a higher level of burden experienced by significant others [27] ***Quality of life:*** Two studies reported how the quality of life of significant others was adversely negatively affected [13, 31]
Financial resources and responsibility	***Resources and responsibilities:*** Significant others described having to work less hours to provide support to the patient while the patient themselves had to give up working, resulting in lower incomes while their expenses would increase due treatment payments [8, 39, 48] ***Comorbidity:*** Two studies reported that the financial impact on significant others may be positively associated with an increase in comorbidity in people with IA [13, 29]
Needs of significant others	***Social support and alone time:*** Two studies described that fulfilling social support needs and having alone time were essential in helping significant others cope with their role [8, 39]. Significant others reported that having support from formal caregivers was not enough to eliminate the burden perceived by the significant others as they continued to provide the emotional support. To alleviate the burden significant other experiences, they themselves needed emotional support from network groups, or peers [8, 39, 48, 50] ***Tailored information:*** Four studies reported that significant others and the person with IA have an unmet need for significant others to be better informed on the disease, symptoms, and treatment [8, 37, 39, 43]. The studies highlighted that the information had to come from HPRs and be tailored to the educational and cultural background of the significant others. One study reported that 25% of significant others did not receive the adequate support and information they needed and would prefer to receive these from the rheumatologist [54]. In one study, the person with IA believed educating significant others would be helpful [43] ***Recognized as important:*** Significant others reported a need for HPRs to recognize significant others as important by including them in consultations and treatment decisions [27, 54]
Interactions between the person with IA and their significant other	***Communicating diagnosis, symptoms, and needs:*** IA patients’ lack of communication was reported as a barrier to receiving the support they needed from their significant others and as leading to insecurity in the significant other regarding their role [35, 41, 42, 49, 50]. A reason for this lack of communication was based on the IA patient’s perception that the significant other would react negatively to the diagnosis or symptoms. One study reported on the significance of good communication in improving better coping of IA [27] ***Relationship:*** IA was reported in some studies to put strain on the relationship due to the significant other not understanding IA [8, 36, 37, 39, 44, 49]. However, other studies reported that relationships might be fortified due to a mutual increased desire to spend time together or navigate the challenges of arthritis together, fostering a sense of closeness within the relationship ***Intimacy:*** Significant others reported experiencing a decrease in physical contact and intimacy as a loss in their relationship, while people with IA did not mention this issue [8, 36, 37, 39, 44, 49]

### RQ 4. Other relevant information

Two studies identified research gaps related to our RQs. These are listed below.

**Table:** 

Areas for new research	***Future studies:*** Two studies reported a need for future intervention studies investigating the effect of supporting depressive significant others’ and the communication and coping strategies used within the relationship to improve IA patients outcomes [24, 27]

## Discussion

This scoping review aimed to provide a comprehensive overview of the research conducted in the last 15 years mapping the content of significant others role, how significant others support may influence to persons with IAs ability to self-manage and the challenges and subsequent needs of significant others when supporting the person with IA. We have provided an overview of the findings relevant to our four research questions, with the goal of evaluating the value of including and supporting significant others when performing interventions improving people with IAs self-management ability. The results revealed a global and ongoing series of studies conducted over the past 15 years utilizing both qualitative and quantitative designs. This diversity in design underscores the need for future quality assessments of all identified studies and qualitative syntheses, particularly for qualitative interview studies. This could improve our understanding of the validity of these studies reporting. Our identification of only one randomized controlled trial focused on supporting significant others to people with IA, highlights the current lack of such trials testing the impact of this inclusion on self-management interventions.

This scoping review cannot confidently testify to the accuracy of the data reported in the included studies, as we did not perform a critical appraisal of them. Consequently, the reader should only use the findings to understand the scope of the research conducted, and not blindly trust the included study’s findings, reported in this review. This review has some limitations. First, since “significant other” is a broad term, which includes many different terminologies, there is a possibility we may have missed some terms applied within our searches leading to relevant studies not being identified in the search. Secondly, we limited our search in terms of language and time, which may have also resulted in the exclusion of relevant studies; however, this limitation was necessary due to the large number of hits obtained. Last, as the studies investigated different types of significant others and the type of significant others in the majority of the included studies was not reported, the team could not determine whether certain element of the significant others role, influences, challenges and needs were more or less frequently reported among the different types of significant others (spouse, parent, adult child, etc.). Despite these limitations, we have conducted a scoping review according to the well-established JBI methodology [9] thus ensuring a thoroughness in the systematic review. According to JBI, conducting the large search, systematic screening, and extraction and applying the content analysis ensured the systematic categorization and reporting of the findings and the overall validity of the results in this review.

The current review identified studies reporting on the substantial supportive role undertaken by significant others to people with IA in their daily self-management of IA. Our findings align with existing systematic reviews on the role of the family in chronic illness management, suggesting that the significant others of people with IA provide support equal to that of the significant others of people with other chronic illnesses like diabetes [57, 58].

Significant others were found to influence the self-management abilities of individuals with IA both positively and negatively. This is in accordance with existing literature on the influence of significant others in chronic disease management and highlights the need for interventions applying a family-system-theory approach to ensure that individuals with IA and their significant others approach disease management positively, with significant others providing support to the person with IA rather than hindering their effective self-management [59, 60]. This includes HPRs helping significant others to employ positive and effective support efforts towards the person with IA [60].

The emotional, financial and relationship challenges experienced by significant others in the included studies of this review have also been identified in other reviews. A review from 2013 investigating the impact on significant others to people with various chronic illnesses, identified key areas where the significant others was negatively impacted to be: psychological distress, financial well-being, relationships, social life, and leisure time [61]. Results who are very similar to our findings. In this scoping review we found that the significant others to people with IA expressed a need for information, involvement, support, and recognition. Although communication and collaboration were not explicitly reported as needs from significant others or the people with IA, our findings suggested that both factors were crucial for the person with IA and their significant others to self-manage IA together. Family-system-theory or dyad coping theories can help in understanding how HPRs can effectively support the coping abilities of both parties. According to both theories, HPR must recognize that interventions targeting self-management in people with IAs cannot be limited to only the person with IA. Instead, they should incorporate the significant others’ supportive role to people with IA, consider the significant others own emotional management enabling the significant other to provide support, as well as the communicative and collaborative strategies of both the person with IA and the significant other [59, 62]. In summary, from a theoretical standpoint, family-system theory and dyadic coping approaches seem to also be relevant within the rheumatology field.

In the review, only one randomized controlled trial (RCT) aimed at supporting significant others was identified, however this study did not apply a family-system-theory or dyadic coping approach, not did it support the person with IA and the significant other together, but solely focused on the significant others [25]. Due to the lack of available RCTs investigating the effect of interventions based on family-system-theory or dyadic coping approaches, we do not know whether such interventions would prove superior to current standards. Therefore, future research should aim to develop interventions aiming at improving people with IA and their significant others self-management ability based on the results from the identified studies, which can be tested in clinical rheumatology practice.

Given the lack of family-system-theory self-management interventions within the rheumatology field, it may be necessary to examine such interventions from other disease areas. Examples include couple-oriented interventions for chronic illness, family interventions for diabetes [59, 63] and models for mobilizing significant others’ support for chronic disease management [57]. These interventions include training significant others in supportive communication and coping techniques to help motivate the diagnosed person in meeting behavioral goals, communicate openly about symptoms, and engage in self-management using cognitive-behavioral-therapy techniques [57].

We conclude that, like other chronic disease settings, the significant others of people with IA take on significant responsibility in relation to the self-management of IA as well as report challenges with this role. Our findings suggest that the significant other’s role and needs are not currently recognized nor met within clinical rheumatology care. Considering this, both people with IA and their significant others could benefit from a cultural shift towards viewing these two parties as one unit. Future RCTs are needed to confirm whether self-management interventions targeting both the person with IA and their significant other, particularly their communicative and collaborative skills, can prove more effective than current self-management interventions solely targeting the person with IA.

## Supplementary Information

Below is the link to the electronic supplementary material.

Supplementary file1 (DOCX 1038 kb)
