# Supplementary material for: Significant others in inflammatory arthritis: roles, influences, and challenges—a scoping review
Source: Rheumatol Int. 2024 Jul 6;44(10):1849–59. doi: 10.1007/s00296-024-05639-9 (PMC11393152; doi:10.1007/s00296-024-05639-9)
Supplement: Supplementary file 1 — Supplementary file1 (DOCX 1038 kb) [file 296_2024_5639_MOESM1_ESM.docx]

# Supplementary File 1. Search strategy

| Participant | Concept | Context |
| --- | --- | --- |
| “Significant other*”  Relative*  “Next of kin”  “Care network member*”  Partner*  Spouse*  Wife  Wives  Husband*  “Other half*”  “Adult child*”  Famil*  “Social Support”  AND  IA  Inflammatory arthritis  RA  Rheumatoid arthritis  PsA  Psoriatic arthritis  SpA  Spondylo arthritis  Spondylitis  Spondylarthritis  Morbus bechterew | Role*  Responsibil*  Impact*  Experience*  Perspective*  View* |  |

# Supplementary file 2: Database searches

| **Search MEDLINE (PubMed) 14-04-2024 2.342 hits -update** |
| --- |
| 1,"(""role*""[All Fields] OR ""responsibil*""[All Fields] OR ""impact*""[All Fields] OR ""experience*""[All Fields] OR ""need*""[All Fields] OR ""perspective*""[All Fields] OR ""view*""[All Fields]) AND (""IA""[All Fields] OR ""Inflammatory arthritis""[All Fields] OR ""RA""[All Fields] OR ""Rheumatoid Arthritis""[All Fields] OR ""PsA""[All Fields] OR ""psoriatic arthritis""[All Fields] OR ""SpA""[All Fields] OR ""Spondylo arthritis""[All Fields] OR (""spondylitis""[MeSH Terms] OR ""spondylitis""[All Fields] OR ""spondylitides""[All Fields]) OR (""spondylarthritis""[MeSH Terms] OR ""spondylarthritis""[All Fields] OR ""spondylarthritides""[All Fields]) OR ""morbus becht*""[All Fields]) AND (""relative*""[All Fields] OR ""Next of kin""[All Fields] OR ""significant other*""[All Fields] OR ""care network member*""[All Fields] OR ""partner*""[All Fields] OR ""spouse*""[All Fields] OR (""spouses""[MeSH Terms] OR ""spouses""[All Fields] OR ""wife""[All Fields]) OR (""spouses""[MeSH Terms] OR ""spouses""[All Fields] OR ""wives""[All Fields]) OR ""parent*""[All Fields] OR ""mother*""[All Fields] OR ""farther*""[All Fields] OR ""social support""[All Fields] OR ""husband*""[All Fields] OR ""other half*""[All Fields] OR ""adult child*""[All Fields] OR ""famil*""[All Fields]) AND (2007/01/01:3000/12/12[Date - Publication] AND (""danish""[Language] OR ""english""[Language] OR ""norwegian""[Language] OR ""swedish""[Language]))",Most Recent,"Danish, English, Norwegian, Swedish, from 2023 - 2024","((""role*""[All Fields] OR ""responsibil*""[All Fields] OR ""impact*""[All Fields] OR ""experience*""[All Fields] OR ""need*""[All Fields] OR ""perspective*""[All Fields] OR ""view*""[All Fields]) AND (""IA""[All Fields] OR ""Inflammatory arthritis""[All Fields] OR ""RA""[All Fields] OR ""Rheumatoid Arthritis""[All Fields] OR ""PsA""[All Fields] OR ""psoriatic arthritis""[All Fields] OR ""SpA""[All Fields] OR ""Spondylo arthritis""[All Fields] OR (""spondylitis""[MeSH Terms] OR ""spondylitis""[All Fields] OR ""spondylitides""[All Fields]) OR (""spondylarthritis""[MeSH Terms] OR ""spondylarthritis""[All Fields] OR ""spondylarthritides""[All Fields]) OR ""morbus becht*""[All Fields]) AND (""relative*""[All Fields] OR ""Next of kin""[All Fields] OR ""significant other*""[All Fields] OR ""care network member*""[All Fields] OR ""partner*""[All Fields] OR ""spouse*""[All Fields] OR (""spouses""[MeSH Terms] OR ""spouses""[All Fields] OR ""wife""[All Fields]) OR (""spouses""[MeSH Terms] OR ""spouses""[All Fields] OR ""wives""[All Fields]) OR ""parent*""[All Fields] OR ""mother*""[All Fields] OR ""farther*""[All Fields] OR ""social support""[All Fields] OR ""husband*""[All Fields] OR ""other half*""[All Fields] OR ""adult child*""[All Fields] OR ""famil*""[All Fields]) AND (2007/01/01:3000/12/12[Date - Publication] AND (""danish""[Language] OR ""english""[Language] OR ""norwegian""[Language] OR ""swedish""[Language]))) AND ((danish[Filter] OR english[Filter] OR norwegian[Filter] OR swedish[Filter]) AND (2023:2024[pdat]))","2,342",23:46:40 |
| **Search MEDLINE (PubMed) 21-02-2023 14,450 hits – original search** |
| 49,"((((((((role*) OR (responsibil*)) OR (impact*)) OR (experience*)) OR (need*)) OR (perspective*)) OR (view*)) AND (((((((((((IA) OR (""Inflammatory arthritis"")) OR (RA)) OR (""Rheumatoid Arthritis"")) OR (PsA)) OR (""psoriatic arthritis"")) OR (SpA)) OR (""Spondylo arthritis"")) OR (Spondylitis)) OR (Spondylarthritis)) OR (""Morbus Becht*""))) AND ((((((((((((relative*) OR (""Next of kin"")) OR (""significant other*"")) OR (""care network member*"")) OR (partner*)) OR (Spouse*)) OR (wife)) OR (wives)) OR (husband*)) OR (""other half*"")) OR (""adult child*"")) OR (famil*))",,"Danish, English, Norwegian, Swedish, from 2007/1/1 - 3000/12/12","((""role*""[All Fields] OR ""responsibil*""[All Fields] OR ""impact*""[All Fields] OR ""experience*""[All Fields] OR ""need*""[All Fields] OR ""perspective*""[All Fields] OR ""view*""[All Fields]) AND (""IA""[All Fields] OR ""Inflammatory arthritis""[All Fields] OR ""RA""[All Fields] OR ""Rheumatoid Arthritis""[All Fields] OR ""PsA""[All Fields] OR ""psoriatic arthritis""[All Fields] OR ""SpA""[All Fields] OR ""Spondylo arthritis""[All Fields] OR (""spondylitis""[MeSH Terms] OR ""spondylitis""[All Fields] OR ""spondylitides""[All Fields]) OR (""spondylarthritis""[MeSH Terms] OR ""spondylarthritis""[All Fields] OR ""spondylarthritides""[All Fields]) OR ""morbus becht*""[All Fields]) AND (""relative*""[All Fields] OR ""Next of kin""[All Fields] OR ""significant other*""[All Fields] OR ""care network member*""[All Fields] OR ""partner*""[All Fields] OR ""spouse*""[All Fields] OR (""spouses""[MeSH Terms] OR ""spouses""[All Fields] OR ""wife""[All Fields]) OR (""spouses""[MeSH Terms] OR ""spouses""[All Fields] OR ""wives""[All Fields]) OR ""husband*""[All Fields] OR ""other half*""[All Fields] OR ""adult child*""[All Fields] OR ""famil*""[All Fields])) AND ((2007/1/1:3000/12/12[pdat]) AND (danish[Filter] OR english[Filter] OR norwegian[Filter] OR swedish[Filter]))","14,450",09:02:24 |
| 44,"((((((((role*) OR (responsibil*)) OR (impact*)) OR (experience*)) OR (need*)) OR (perspective*)) OR (view*)) AND (((((((((((IA) OR (""Inflammatory arthritis"")) OR (RA)) OR (""Rheumatoid Arthritis"")) OR (PsA)) OR (""psoriatic arthritis"")) OR (SpA)) OR (""Spondylo arthritis"")) OR (Spondylitis)) OR (Spondylarthritis)) OR (""Morbus Becht*""))) AND ((((((((((((relative*) OR (""Next of kin"")) OR (""significant other*"")) OR (""care network member*"")) OR (partner*)) OR (Spouse*)) OR (wife)) OR (wives)) OR (husband*)) OR (""other half*"")) OR (""adult child*"")) OR (famil*))",,from 2007/1/1 - 3000/12/12,"((""role*""[All Fields] OR ""responsibil*""[All Fields] OR ""impact*""[All Fields] OR ""experience*""[All Fields] OR ""need*""[All Fields] OR ""perspective*""[All Fields] OR ""view*""[All Fields]) AND (""IA""[All Fields] OR ""Inflammatory arthritis""[All Fields] OR ""RA""[All Fields] OR ""Rheumatoid Arthritis""[All Fields] OR ""PsA""[All Fields] OR ""psoriatic arthritis""[All Fields] OR ""SpA""[All Fields] OR ""Spondylo arthritis""[All Fields] OR (""spondylitis""[MeSH Terms] OR ""spondylitis""[All Fields] OR ""spondylitides""[All Fields]) OR (""spondylarthritis""[MeSH Terms] OR ""spondylarthritis""[All Fields] OR ""spondylarthritides""[All Fields]) OR ""morbus becht*""[All Fields]) AND (""relative*""[All Fields] OR ""Next of kin""[All Fields] OR ""significant other*""[All Fields] OR ""care network member*""[All Fields] OR ""partner*""[All Fields] OR ""spouse*""[All Fields] OR (""spouses""[MeSH Terms] OR ""spouses""[All Fields] OR ""wife""[All Fields]) OR (""spouses""[MeSH Terms] OR ""spouses""[All Fields] OR ""wives""[All Fields]) OR ""husband*""[All Fields] OR ""other half*""[All Fields] OR ""adult child*""[All Fields] OR ""famil*""[All Fields])) AND (2007/1/1:3000/12/12[pdat])","12,232",08:47:49 |
| 43,"((((((((role*) OR (responsibil*)) OR (impact*)) OR (experience*)) OR (need*)) OR (perspective*)) OR (view*)) AND (((((((((((IA) OR (""Inflammatory arthritis"")) OR (RA)) OR (""Rheumatoid Arthritis"")) OR (PsA)) OR (""psoriatic arthritis"")) OR (SpA)) OR (""Spondylo arthritis"")) OR (Spondylitis)) OR (Spondylarthritis)) OR (""Morbus Becht*""))) AND ((((((((((((relative*) OR (""Next of kin"")) OR (""significant other*"")) OR (""care network member*"")) OR (partner*)) OR (Spouse*)) OR (wife)) OR (wives)) OR (husband*)) OR (""other half*"")) OR (""adult child*"")) OR (famil*))",,,"(""role*""[All Fields] OR ""responsibil*""[All Fields] OR ""impact*""[All Fields] OR ""experience*""[All Fields] OR ""need*""[All Fields] OR ""perspective*""[All Fields] OR ""view*""[All Fields]) AND (""IA""[All Fields] OR ""Inflammatory arthritis""[All Fields] OR ""RA""[All Fields] OR ""Rheumatoid Arthritis""[All Fields] OR ""PsA""[All Fields] OR ""psoriatic arthritis""[All Fields] OR ""SpA""[All Fields] OR ""Spondylo arthritis""[All Fields] OR (""spondylitis""[MeSH Terms] OR ""spondylitis""[All Fields] OR ""spondylitides""[All Fields]) OR (""spondylarthritis""[MeSH Terms] OR ""spondylarthritis""[All Fields] OR ""spondylarthritides""[All Fields]) OR ""morbus becht*""[All Fields]) AND (""relative*""[All Fields] OR ""Next of kin""[All Fields] OR ""significant other*""[All Fields] OR ""care network member*""[All Fields] OR ""partner*""[All Fields] OR ""spouse*""[All Fields] OR (""spouses""[MeSH Terms] OR ""spouses""[All Fields] OR ""wife""[All Fields]) OR (""spouses""[MeSH Terms] OR ""spouses""[All Fields] OR ""wives""[All Fields]) OR ""husband*""[All Fields] OR ""other half*""[All Fields] OR ""adult child*""[All Fields] OR ""famil*""[All Fields])","16,806",08:47:21 |
| 42,((((((role*) OR (responsibil*)) OR (impact*)) OR (experience*)) OR (need*)) OR (perspective*)) OR (view*),,,"""role*""[All Fields] OR ""responsibil*""[All Fields] OR ""impact*""[All Fields] OR ""experience*""[All Fields] OR ""need*""[All Fields] OR ""perspective*""[All Fields] OR ""view*""[All Fields]","7,741,098",08:45:54 |
| 41,view*,,,"""view*""[All Fields]","544,535",08:45:32 |
| 40,perspective*,,,"""perspective*""[All Fields]","408,005",08:45:15 |
| 38,need*,,,"""need*""[All Fields]","2,221,753",08:39:13 |
| 35,experience*,,,"""experience*""[All Fields]","1,245,198",08:38:32 |
| 34,impact*,,,"""impact*""[All Fields]","1,399,634",08:38:15 |
| 31,responsibil*,,,"""responsibil*""[All Fields]","101,390",08:37:42 |
| 27,role*,,,"""role*""[All Fields]","3,366,339",08:37:09 |
| 26,"((((((((((IA) OR (""Inflammatory arthritis"")) OR (RA)) OR (""Rheumatoid Arthritis"")) OR (PsA)) OR (""psoriatic arthritis"")) OR (SpA)) OR (""Spondylo arthritis"")) OR (Spondylitis)) OR (Spondylarthritis)) OR (""Morbus Becht*"")",,,"""IA""[All Fields] OR ""Inflammatory arthritis""[All Fields] OR ""RA""[All Fields] OR ""Rheumatoid Arthritis""[All Fields] OR ""PsA""[All Fields] OR ""psoriatic arthritis""[All Fields] OR ""SpA""[All Fields] OR ""Spondylo arthritis""[All Fields] OR ""spondylitis""[MeSH Terms] OR ""spondylitis""[All Fields] OR ""spondylitides""[All Fields] OR ""spondylarthritis""[MeSH Terms] OR ""spondylarthritis""[All Fields] OR ""spondylarthritides""[All Fields] OR ""morbus becht*""[All Fields]","417,776",08:36:56 |
| 25,"""Morbus Becht*""",,,"""morbus becht*""[All Fields]",189,08:36:21 |
| 24,Spondylarthritis,,,"""spondylarthritis""[MeSH Terms] OR ""spondylarthritis""[All Fields] OR ""spondylarthritides""[All Fields]","29,395",08:35:57 |
| 23,Spondylitis,,,"""spondylitis""[MeSH Terms] OR ""spondylitis""[All Fields] OR ""spondylitides""[All Fields]","42,880",08:35:21 |
| 22,"""Spondylo arthritis""",,,"""Spondylo arthritis""[All Fields]",32,08:34:50 |
| 21,SpA,,,"""SpA""[All Fields]","23,313",08:34:33 |
| 20,"""psoriatic arthritis""",,,"""psoriatic arthritis""[All Fields]","12,116",08:34:23 |
| 19,PsA,,,"""PsA""[All Fields]","40,902",08:33:54 |
| 18,"""Rheumatoid Arthritis""",,,"""Rheumatoid Arthritis""[All Fields]","145,087",08:33:48 |
| 17,RA,,,"""RA""[All Fields]","111,035",08:33:31 |
| 16,"""Inflammatory arthritis""",,,"""Inflammatory arthritis""[All Fields]","5,707",08:33:27 |
| 15,IA,,,"""IA""[All Fields]","117,402",08:33:11 |
| 14,"(((((((((((relative*) OR (""Next of kin"")) OR (""significant other*"")) OR (""care network member*"")) OR (partner*)) OR (Spouse*)) OR (wife)) OR (wives)) OR (husband*)) OR (""other half*"")) OR (""adult child*"")) OR (famil*)",,,"""relative*""[All Fields] OR ""Next of kin""[All Fields] OR ""significant other*""[All Fields] OR ""care network member*""[All Fields] OR ""partner*""[All Fields] OR ""spouse*""[All Fields] OR ""spouses""[MeSH Terms] OR ""spouses""[All Fields] OR ""wife""[All Fields] OR ""spouses""[MeSH Terms] OR ""spouses""[All Fields] OR ""wives""[All Fields] OR ""husband*""[All Fields] OR ""other half*""[All Fields] OR ""adult child*""[All Fields] OR ""famil*""[All Fields]","3,298,276",08:33:03 |
| 13,famil*,,,"""famil*""[All Fields]","1,567,384",08:32:08 |
| 12,"""adult child*""",,,"""adult child*""[All Fields]","4,202",08:31:51 |
| 11,"""other half*""",,,"""other half*""[All Fields]","9,508",08:31:27 |
| 10,husband*,,,"""husband*""[All Fields]","51,140",08:31:10 |
| 9,wives,,,"""spouses""[MeSH Terms] OR ""spouses""[All Fields] OR ""wives""[All Fields]","22,988",08:29:58 |
| 8,wife,,,"""spouses""[MeSH Terms] OR ""spouses""[All Fields] OR ""wife""[All Fields]","25,134",08:29:43 |
| 7,Spouse*,,,"""spouse*""[All Fields]","34,885",08:29:29 |
| 6,partner*,,,"""partner*""[All Fields]","277,818",08:29:21 |
| 5,"""care network member*""",,,"""care network member*""[All Fields]",9,08:29:08 |
| 4,"""significant other*""",,,"""significant other*""[All Fields]","4,496",08:27:09 |
| 3,"""Next of kin""",,,"""Next of kin""[All Fields]","1,961",08:26:24 |
| 2,relative*,,,"""relative*""[All Fields]","1,562,967",08:25:02 |

## Search PsycInfo – update 14-04-2024 7 hits


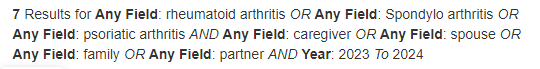


## Search PsycInfo 21-02-2023 150 hits

#
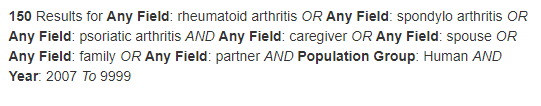


**Search Embase (Ovid) – update- 19-04-2024 79 hits**

**1 "rheumatoid arthritis".mp. [mp=title, abstract, heading word, drug trade name, original title, device manufacturer, drug manufacturer, device trade name, keyword heading word, floating subheading word, candidate term word] 267408**

**2 inflammatory arthritis.mp. [mp=title, abstract, heading word, drug trade name, original title, device manufacturer, drug manufacturer, device trade name, keyword heading word, floating subheading word, candidate term word] 12915**

**3 inflammatory arthritis.mp. or exp rheumatoid arthritis/ 252015**

**4 psoriatic arthritis.mp. [mp=title, abstract, heading word, drug trade name, original title, device manufacturer, drug manufacturer, device trade name, keyword heading word, floating subheading word, candidate term word] 34973**

**5 exp psoriatic arthritis/ 32386**

**6 exp ankylosing spondylitis/ or exp spondylarthritis/ 73679**

**7 morbus bechterew.mp. 193**

**8 1 or 2 or 3 or 4 or 5 or 6 or 7 327634**

**9 significant other.mp. 2276**

**10 exp relative/ 376965**

**11 family/ or caregiver/ 219835**

**12 next of kin.mp. 2799**

**13 exp social network/ or exp social support/ 147062**

**14 exp domestic partner/ 550**

**15 exp spouse/ or spouse.mp. 33530**

**16 wife.mp. or exp wife/ 9635**

**17 husband.mp. or husband/ 11257**

**18 other half.mp. 13051**

**19 adult child.mp. or adult child/ 3086**

**20 9 or 10 or 11 or 12 or 13 or 14 or 15 or 16 or 17 or 18 or 19 744071**

**21 8 and 20 4335**

**22 role.mp. 4131911**

**23 responsibility.mp. or exp responsibility/ 105255**

**24 health impact assessment/ 8742**

**25 experience.mp. or exp personal experience/ or experience/ 1306629**

**26 exp life course perspective/ 453**

**27 perception.mp. or perception/ 458722**

**28 22 or 23 or 24 or 25 or 26 or 27 5747032**

**29 21 and 28 1101**

**30 29 and 2007:2024.(sa_year). 969**

**31 limit 30 to yr="2023 - 2024" 78**

**Search Embase (Ovid) 21-02-2023 883 hits**

Embase <1974 to 2023 February 20>

1 "rheumatoid arthritis".mp. [mp=title, abstract, heading word, drug trade name, original title, device manufacturer, drug manufacturer, device trade name, keyword heading word, floating subheading word, candidate term word] 252679

2 inflammatory arthritis.mp. [mp=title, abstract, heading word, drug trade name, original title, device manufacturer, drug manufacturer, device trade name, keyword heading word, floating subheading word, candidate term word] 12031

3 inflammatory arthritis.mp. or exp rheumatoid arthritis/ 237852

4 psoriatic arthritis.mp. [mp=title, abstract, heading word, drug trade name, original title, device manufacturer, drug manufacturer, device trade name, keyword heading word, floating subheading word, candidate term word] 32203

5 exp psoriatic arthritis/ 29853

6 exp ankylosing spondylitis/ or exp spondylarthritis/ 68612

7 morbus bechterew.mp. 194

8 1 or 2 or 3 or 4 or 5 or 6 or 7 308975

9 significant other.mp. 2141

10 exp relative/ 356914

11 family/ or caregiver/ 200704

12 next of kin.mp. 2609

13 exp social network/ or exp social support/ 131886

14 exp domestic partner/ 424

15 exp spouse/ or spouse.mp. 31095

16 wife.mp. or exp wife/ 9035

17 husband.mp. or husband/ 10457

18 other half.mp. 12430

19 adult child.mp. or adult child/ 2859

20 9 or 10 or 11 or 12 or 13 or 14 or 15 or 16 or 17 or 18 or 19 691366

21 8 and 20 4044

22 role.mp. 3880002

23 responsibility.mp. or exp responsibility/ 98684

24 health impact assessment/ 7265

25 experience.mp. or exp personal experience/ or experience/ 1206192

26 exp life course perspective/ 225

27 perception.mp. or perception/ 427407

28 22 or 23 or 24 or 25 or 26 or 27 5378153

29 21 and 28 1015

30 29 and 2007:2024.(sa_year). 883 hits

Search CINAHL -update- 19-04 2024 8:02:21 AM, 1 hits

| **#** | **Query** | **Limiters/Expanders** | **Last Run Via** | **Results** |
| --- | --- | --- | --- | --- |
| S24 | S21 AND S22 | Limiters_publication Date:20230101-20231231  Expanders - Apply equivalent subjects Search modes - Boolean/Phrase | Interface - EBSCOhost Research Databases Search Screen - Basic Search Database - CINAHL Plus with Full Text | 1 |
| S23 | S21 AND S22 | Expanders - Apply equivalent subjects Search modes - Boolean/Phrase | Interface - EBSCOhost Research Databases Search Screen - Basic Search Database - CINAHL Plus with Full Text | Display |
| S22 | S19 AND S20 | Expanders - Apply equivalent subjects Search modes - Boolean/Phrase | Interface - EBSCOhost Research Databases Search Screen - Basic Search Database - CINAHL Plus with Full Text | Display |
| S21 | S12 OR S13 OR S14 OR S15 OR S16 OR S17 OR S18 | Expanders - Apply equivalent subjects Search modes - Boolean/Phrase | Interface - EBSCOhost Research Databases Search Screen - Basic Search Database - CINAHL Plus with Full Text | Display |
| S20 | S3 OR S4 OR S5 OR S6 OR S7 OR S8 OR S9 OR S10 OR S11 | Expanders - Apply equivalent subjects Search modes - Boolean/Phrase | Interface - EBSCOhost Research Databases Search Screen - Basic Search Database - CINAHL Plus with Full Text | Display |
| S19 | S1 OR S2 | Expanders - Apply equivalent subjects Search modes - Boolean/Phrase | Interface - EBSCOhost Research Databases Search Screen - Basic Search Database - CINAHL Plus with Full Text | Display |
| S18 | (MM "Perception") OR "perception" OR (MM "Psychological Well-Being") | Expanders - Apply equivalent subjects Search modes - Boolean/Phrase | Interface - EBSCOhost Research Databases Search Screen - Basic Search Database - CINAHL Plus with Full Text | Display |
| S17 | view | Expanders - Apply equivalent subjects Search modes - Boolean/Phrase | Interface - EBSCOhost Research Databases Search Screen - Advanced Search Database - CINAHL Plus with Full Text | Display |
| S16 | (MM "Life Course Perspective") OR "perspective" | Expanders - Apply equivalent subjects Search modes - Boolean/Phrase | Interface - EBSCOhost Research Databases Search Screen - Advanced Search Database - CINAHL Plus with Full Text | Display |
| S15 | (MM "Life Experiences") OR "experience" | Expanders - Apply equivalent subjects Search modes - Boolean/Phrase | Interface - EBSCOhost Research Databases Search Screen - Advanced Search Database - CINAHL Plus with Full Text | Display |
| S14 | "impact" | Expanders - Apply equivalent subjects Search modes - Boolean/Phrase | Interface - EBSCOhost Research Databases Search Screen - Advanced Search Database - CINAHL Plus with Full Text | Display |
| S13 | (MM "Social Responsibility") OR "responsibility" | Expanders - Apply equivalent subjects Search modes - Boolean/Phrase | Interface - EBSCOhost Research Databases Search Screen - Advanced Search Database - CINAHL Plus with Full Text | Display |
| S12 | (MM "Role") OR "role" | Expanders - Apply equivalent subjects Search modes - Boolean/Phrase | Interface - EBSCOhost Research Databases Search Screen - Advanced Search Database - CINAHL Plus with Full Text | Display |
| S11 | "husband" | Expanders - Apply equivalent subjects Search modes - Boolean/Phrase | Interface - EBSCOhost Research Databases Search Screen - Advanced Search Database - CINAHL Plus with Full Text | Display |
| S10 | "wife" | Expanders - Apply equivalent subjects Search modes - Boolean/Phrase | Interface - EBSCOhost Research Databases Search Screen - Advanced Search Database - CINAHL Plus with Full Text | Display |
| S9 | (MM "Spouses") OR (MM "Caregivers") OR "spouse" | Expanders - Apply equivalent subjects Search modes - Boolean/Phrase | Interface - EBSCOhost Research Databases Search Screen - Advanced Search Database - CINAHL Plus with Full Text | Display |
| S8 | (MM "Sexual Partners") | Expanders - Apply equivalent subjects Search modes - Boolean/Phrase | Interface - EBSCOhost Research Databases Search Screen - Advanced Search Database - CINAHL Plus with Full Text | Display |
| S7 | (MM "Social Networks") | Expanders - Apply equivalent subjects Search modes - Boolean/Phrase | Interface - EBSCOhost Research Databases Search Screen - Advanced Search Database - CINAHL Plus with Full Text | Display |
| S6 | care network member | Expanders - Apply equivalent subjects Search modes - Boolean/Phrase | Interface - EBSCOhost Research Databases Search Screen - Advanced Search Database - CINAHL Plus with Full Text | Display |
| S5 | "next of kin" | Expanders - Apply equivalent subjects Search modes - Boolean/Phrase | Interface - EBSCOhost Research Databases Search Screen - Advanced Search Database - CINAHL Plus with Full Text | Display |
| S4 | "relatives" | Expanders - Apply equivalent subjects Search modes - Boolean/Phrase | Interface - EBSCOhost Research Databases Search Screen - Advanced Search Database - CINAHL Plus with Full Text | Display |
| S3 | (MM "Significant Other") OR (MM "Family") OR (MM "Adult Children") OR (MM "Family Functioning") OR (MM "Family Relations") | Expanders - Apply equivalent subjects Search modes - Boolean/Phrase | Interface - EBSCOhost Research Databases Search Screen - Advanced Search Database - CINAHL Plus with Full Text | Display |
| S2 | (MM "Arthritis, Rheumatoid") OR (MM "Arthritis, Psoriatic") OR (MM "Ankylosis") OR (MM "Spondylarthritis") OR (MM "Spondylarthropathies") | Expanders - Apply equivalent subjects Search modes - Boolean/Phrase | Interface - EBSCOhost Research Databases Search Screen - Advanced Search Database - CINAHL Plus with Full Text | Display |
| S1 | inflammatory arthritis | Expanders - Apply equivalent subjects Search modes - Boolean/Phrase | Interface - EBSCOhost Research Databases Search Screen - Advanced Search | Display |

Search CINAHL 21-02 2023 4:02:21 PM, 127 hits

| **#** | **Query** | **Limiters/Expanders** | **Last Run Via** | **Results** |
| --- | --- | --- | --- | --- |
| S23 | S21 AND S22 | Expanders - Apply equivalent subjects Search modes - Boolean/Phrase | Interface - EBSCOhost Research Databases Search Screen - Basic Search Database - CINAHL Plus with Full Text | Display |
| S22 | S19 AND S20 | Expanders - Apply equivalent subjects Search modes - Boolean/Phrase | Interface - EBSCOhost Research Databases Search Screen - Basic Search Database - CINAHL Plus with Full Text | Display |
| S21 | S12 OR S13 OR S14 OR S15 OR S16 OR S17 OR S18 | Expanders - Apply equivalent subjects Search modes - Boolean/Phrase | Interface - EBSCOhost Research Databases Search Screen - Basic Search Database - CINAHL Plus with Full Text | Display |
| S20 | S3 OR S4 OR S5 OR S6 OR S7 OR S8 OR S9 OR S10 OR S11 | Expanders - Apply equivalent subjects Search modes - Boolean/Phrase | Interface - EBSCOhost Research Databases Search Screen - Basic Search Database - CINAHL Plus with Full Text | Display |
| S19 | S1 OR S2 | Expanders - Apply equivalent subjects Search modes - Boolean/Phrase | Interface - EBSCOhost Research Databases Search Screen - Basic Search Database - CINAHL Plus with Full Text | Display |
| S18 | (MM "Perception") OR "perception" OR (MM "Psychological Well-Being") | Expanders - Apply equivalent subjects Search modes - Boolean/Phrase | Interface - EBSCOhost Research Databases Search Screen - Basic Search Database - CINAHL Plus with Full Text | Display |
| S17 | view | Expanders - Apply equivalent subjects Search modes - Boolean/Phrase | Interface - EBSCOhost Research Databases Search Screen - Advanced Search Database - CINAHL Plus with Full Text | Display |
| S16 | (MM "Life Course Perspective") OR "perspective" | Expanders - Apply equivalent subjects Search modes - Boolean/Phrase | Interface - EBSCOhost Research Databases Search Screen - Advanced Search Database - CINAHL Plus with Full Text | Display |
| S15 | (MM "Life Experiences") OR "experience" | Expanders - Apply equivalent subjects Search modes - Boolean/Phrase | Interface - EBSCOhost Research Databases Search Screen - Advanced Search Database - CINAHL Plus with Full Text | Display |
| S14 | "impact" | Expanders - Apply equivalent subjects Search modes - Boolean/Phrase | Interface - EBSCOhost Research Databases Search Screen - Advanced Search Database - CINAHL Plus with Full Text | Display |
| S13 | (MM "Social Responsibility") OR "responsibility" | Expanders - Apply equivalent subjects Search modes - Boolean/Phrase | Interface - EBSCOhost Research Databases Search Screen - Advanced Search Database - CINAHL Plus with Full Text | Display |
| S12 | (MM "Role") OR "role" | Expanders - Apply equivalent subjects Search modes - Boolean/Phrase | Interface - EBSCOhost Research Databases Search Screen - Advanced Search Database - CINAHL Plus with Full Text | Display |
| S11 | "husband" | Expanders - Apply equivalent subjects Search modes - Boolean/Phrase | Interface - EBSCOhost Research Databases Search Screen - Advanced Search Database - CINAHL Plus with Full Text | Display |
| S10 | "wife" | Expanders - Apply equivalent subjects Search modes - Boolean/Phrase | Interface - EBSCOhost Research Databases Search Screen - Advanced Search Database - CINAHL Plus with Full Text | Display |
| S9 | (MM "Spouses") OR (MM "Caregivers") OR "spouse" | Expanders - Apply equivalent subjects Search modes - Boolean/Phrase | Interface - EBSCOhost Research Databases Search Screen - Advanced Search Database - CINAHL Plus with Full Text | Display |
| S8 | (MM "Sexual Partners") | Expanders - Apply equivalent subjects Search modes - Boolean/Phrase | Interface - EBSCOhost Research Databases Search Screen - Advanced Search Database - CINAHL Plus with Full Text | Display |
| S7 | (MM "Social Networks") | Expanders - Apply equivalent subjects Search modes - Boolean/Phrase | Interface - EBSCOhost Research Databases Search Screen - Advanced Search Database - CINAHL Plus with Full Text | Display |
| S6 | care network member | Expanders - Apply equivalent subjects Search modes - Boolean/Phrase | Interface - EBSCOhost Research Databases Search Screen - Advanced Search Database - CINAHL Plus with Full Text | Display |
| S5 | "next of kin" | Expanders - Apply equivalent subjects Search modes - Boolean/Phrase | Interface - EBSCOhost Research Databases Search Screen - Advanced Search Database - CINAHL Plus with Full Text | Display |
| S4 | "relatives" | Expanders - Apply equivalent subjects Search modes - Boolean/Phrase | Interface - EBSCOhost Research Databases Search Screen - Advanced Search Database - CINAHL Plus with Full Text | Display |
| S3 | (MM "Significant Other") OR (MM "Family") OR (MM "Adult Children") OR (MM "Family Functioning") OR (MM "Family Relations") | Expanders - Apply equivalent subjects Search modes - Boolean/Phrase | Interface - EBSCOhost Research Databases Search Screen - Advanced Search Database - CINAHL Plus with Full Text | Display |
| S2 | (MM "Arthritis, Rheumatoid") OR (MM "Arthritis, Psoriatic") OR (MM "Ankylosis") OR (MM "Spondylarthritis") OR (MM "Spondylarthropathies") | Expanders - Apply equivalent subjects Search modes - Boolean/Phrase | Interface - EBSCOhost Research Databases Search Screen - Advanced Search Database - CINAHL Plus with Full Text | Display |
| S1 | inflammatory arthritis | Expanders - Apply equivalent subjects Search modes - Boolean/Phrase | Interface - EBSCOhost Research Databases Search Screen - Advanced Search | Display |

Search Scopus 19-04-2024 2,886 hits

( TITLE-ABS-KEY ( relative* OR "significant other*" OR caregiver* OR partner* OR spouse* OR famil* OR "social support" ) AND TITLE-ABS-KEY ( "inflammatory arthritis" OR "rheumatoid arthritis" OR "psoriatic arthritis" OR spondylitis OR spondylarthritis OR "morbus bechterew" ) AND TITLE-ABS-KEY ( role* OR responsibil* OR impact* OR experience* OR perspective* OR view* ) ) AND (LIMIT TO (PBLICATION YEAR “2007”-“2024”) OR ( LIMIT-TO ( LANGUAGE , "english" ) OR LIMIT-TO ( LANGUAGE , "danish" ) OR LIMIT-TO ( LANGUAGE , "swedish" ) OR LIMIT-TO ( LANGUAGE , "norwegian" ) OR LIMIT-TO ( LANGUAGE , "icelandic" ) ) AND ( LIMIT-TO ( SUBJAREA , "medi" ) OR LIMIT-TO ( SUBJAREA , "nurs" ) OR LIMIT-TO ( SUBJAREA , "psyc" ) OR LIMIT-TO ( SUBJAREA , "heal" ) OR LIMIT-TO ( SUBJAREA , "mult" ) OR LIMIT-TO ( SUBJAREA , "soci" ) )

# Supplementary File 3. Data extraction tool

|  | |
| --- | --- |
| Scoping review title | Mapping research investigating significant others to people with inflammatory arthritis and their role, influence, challenges, and needs related to disease management: A scoping review |
| Review objective/s | The aim of this scoping review is to identify and map the current research regarding the role and needs of the significant others of people diagnosed with IA, the positive/negative impacts of this role, and current knowledge gaps regarding this role. |
| Review question/s | RQ1. What has been reported on what the role of significant others to people with IA entail, from the respective perspectives of the patient, the significant other, and HPR?  RQ2. What has been reported on significant others influence on people with IAs self-management abilities?  RQ3. What has been reported on the challenges and resultant needs of these significant others?  RQ4. What are the research gaps in the literature regarding significant others to people with IA? |
| **Inclusion/exclusion criteria** | |
| Population | ≥ 18 years of age and diagnosed with rheumatoid arthritis, psoriatic arthritis, or spondyloarthritis (including axial spondyloarthritis and morbus Bechterew); or their ≥ 18 year-old significant other (partner, spouse, family, children, parents, colleagues, neighbors, and friends); or a healthcare professional working with people with inflammatory arthritis |
| Concept | The role of the significant other and the positive/negative impact of this role on both the person with IA and the significant other themselves |
| Context | Studies before 2007 will be excluded |
| Types of evidence sources | Randomized controlled trials, non-randomized controlled trials, before-and-after studies, interrupted time-series studies, prospective and retrospective cohort studies, case-control studies and analytical cross-sectional studies, case series, people case reports, descriptive cross-sectional studies, and qualitative studies will be included. Experimental studies lacking outcomes regarding significant others will be excluded.  In addition, protocols, conference abstracts, and systematic reviews and metanalyses will also be excluded. |
| **Evidence source details and characteristics** | |
| Citation details (e.g., author/s, date, title, journal, volume, issue, pages) |  |
| Country |  |
| Design |  |
| Participant details (e.g., age/sex, number, and role [patient, significant other, or healthcare professional]) |  |
| Setting |  |
| Outcome |  |
| Finding/themes/categories |  |
| Author main conclusion |  |
| **Findings extracted from sources of evidence**(in relation to the concept of the scoping review) | |
| The role of significant others (i.e., what does it entail?) |  |
| Negative impact of the role on people |  |
| Positive impact of the role on people |  |
| Negative impact of the role on significant others and their needs |  |
| Positive impact of the role on significant others and their needs |  |
| Other |  |

# Supplementary File 4. Example of open coding of extracted data and category development

| **RQ1: WHAT IS THE ROLE OF SIGNIFICANT OTHERS?** | | | |
| --- | --- | --- | --- |
| Extracted content | Condensed meaning | Code and sub-categories | Category |
| Relatives also could have numerous roles in medical care. They could take part in medical decisions, be present during consultations, help with adherence to treatment, help in searching for information, provide or search for some assistance with treatment administration, or be a counselor. She helps me do the injection; I prepare the injection, she gives [the injection] to me and checks whether the liquid is not altered. She gives me the alcohol [and] the pad (P21). (Brignon 2020)  If it were up to me, I would have even given up taking medication and visiting doctors. I was very disappointed, but my parents tried very hard to get me out of this state. They booked doctors’ appointments on time and took care of my medication. They supported me in every way. I really owe my current good mood to my parents (Female, single, 22 years old). (Jahani 2022) | Injecting medicine, booking appointments, driving to the doctor, search for information | Medical care | Practical support |
| Examples of activities where the significant others needed to help were opening lids, house cleaning and cutting up food. The participants reported grocery shopping to be a common activity where assistance was needed, and in particular carrying shopping bags. Lifting heavy objects, such as furniture, was also usually problematic. Some participants also mentioned problems with self-care, such as personal hygiene, or dressing. (Bergstrøm 2020) | Opening lids, cutting food, grocery shopping, lifting heavy objects, dressing | Activities of daily living |  |
| Male participants bought over-the-counter medications, paid for transportation to and from medical appointments, hired helpers to maintain the house, and supported their wives’ early retirement decisions. (Fallatah 2015) | Paying for help, medication, early retirement | Financial support |  |
| Fear, anxiety, and despair were ruining me. I avoided everyone. I would not leave the house. I did not want anyone to set foot in our home. My husband talked to me a lot at this stage. When I got better, he persuaded me to go out.  The participant explained that to maintain their marriage she has to motivate her husband and positively influence him and make him feel strong. (Jahani 2022) | Fear, anxiety, and despair ruining me, husband talked to me a lot | Dealing with feelings | Emotional support |
| They talk to me to help me forget some of my pain and discomfort. (Jahani 2022) | Talking to distract from pain and discomfort | Dealing with symptoms |  |
| . . . providing support during minor procedures. Being present, either with the relative in the doctor’s clinic or waiting outside in the waiting area, was a commonly expressed example of emotional support. (Fallatah 2015) | Providing emotional support during doctor clinic visits | Medical care |  |
| Several participants described their assistance in household tasks and changes in the role of the family.  Katiana described her reaction to her mother’s flare ups . . . as well as assuming her mother’s duties toward herself and her sisters. (Fallatah 2015) | Changes in the role of the family, assuming mother’s duties | Taking on former roles of the patient | More than support |
| For some partners, the carer role extended beyond providing daily support, progressing towards a protector role by managing moods and disease flare-ups, which required constant attention. This sense of responsibility to protect was suggestive of an inequitable relationship at times, with the individuals’ with axSpA perceived as vulnerable. (Raybone 2019)  Many participants felt that they had to protect their spouse, and many talked about being overprotective at times by attempting to shield their spouse from emotional and physical distress. (Matheson 2010) | Caretaker role extending beyond support towards protector role, keeping mood elevated, feeling overprotective, shielding spouse from emotional and physical distress | Becoming protectors of the patient |  |

# Supplementary File 5. Final framework

| **Research Questions** | **Category** | **Sub-category** |
| --- | --- | --- |
| RQ 1. The role of significant others | Practical support | ***Activities of daily living***  ***Medical care***  ***Financial aid*** |
|  | Emotional support | ***Helping patients deal with feelings, including depression and anxiety***  ***Medical care***  ***Most important*** |
|  | More than support | ***Taking on a motherly role***  ***(Over)protecting*** |
|  | | |
| RQ 2. Significant others’ influence on people with IA | Disease management | ***Disease activity***  ***Pain***  ***Disability***  ***Treatment adherence***  ***Help-seeking behavior***  ***Self-efficacy*** |
|  | Emotional management | ***Anxiety, depression, and stress***  ***Quality of life*** |
|  | Role management | ***Identity and life roles***  ***Acceptance***  ***Sickness-related absence*** |
|  | | |
| RQ 3. Challenges faced by significant others | Life revolving around the person with IA | ***Loss of social network***  ***Part of family life*** |
|  | Emotional and psychological impact | ***Initial emotional reactions***  ***Emotions related to IA pain***  ***Emotional overload***  ***Depression and stress***  ***Quality of life*** |
|  | Financial resources and responsibility | ***Resources and responsibilities***  ***Comorbidity*** |
|  | Needs of significant others | ***Social support and alone time***  ***Tailored information***  ***Recognized as important*** |
|  | Interactions between the person with IA and significant other | ***Communicating diagnosis, symptoms, and needs***  ***Relationship***  ***Intimacy*** |
|  |  |  |
| RQ 4. Research gaps | Areas for new research | ***Future studies*** |

# Supplementary file 6. Included studies

| Study ID Continent | | Study design | Aim | | | | Participant characteristics | | Outcomes | | Type of significant others | | Author conclusion | | |  |  |
| --- | --- | --- | --- | --- | --- | --- | --- | --- | --- | --- | --- | --- | --- | --- | --- | --- | --- |
| Studies applying a quantitative design, presented in alphabetical order | | | | | | | | | | | | | | | |  |  |
| Benka 2012  Europe | | Cohort study | Psychological distress in RA people and associations between social support and psychological distress. | | | | 116 people with RA, Mean 47.59 years +/- 12.35 SD  85% female | | Joint tenderness  Disease activity  Pain  Functional disability  Social support  Psychological distress | | Social support* | | Initial psychological distress, which was found to have the highest correlation with psychological distress experienced 4 years later. Emotional support and lower pain were associated with lower levels of psychological distress. | | |  |  |
| Bergstrøm (March) 2021, Europe | | Cohort study | How support from significant others affects the associations between disease activity, activity limitations, pain intensity, and grip force in people with RA. | | | | 274 people with RA  Mean age 50 years +/-11 SD  73% female | | Disease  Perceived support  Sick-ness absence | | Wife  Husband Partner  Friend | | Associations between disease activity and sickness absence change with the amount of perceived support from significant others | | |  |  |
| Brandstetter 2017  Europe | | Cross sectional study | Role of social support on the association between pain and depressive symptoms among people with RA. | | | | 361 people with RA  mean 60.2 years +/- 13.36 SD  69% females | | Pain  Social support Depression | | Social support* | | Social support is associated with beneficial health effects, but it did not buffer the negative effects of pain. | | |  |  |
| Cepukiene 2024 Europe | | Cross sectional study | The objective of the present study was to assess the significance of pain self-efficacy and emotional support from a partner in relation to pain severity among women with RA | | | | 205 RA people  Mean age 41.54 years +/- 6.6 SD  100% female | | Pain  Communication  Pain self-efficacy | | Partner | | The study confirms the heterogeneous nature of RA pain, emphasizing the importance of using assessment tools that capture its various components during the treatment process. Given the detrimental impact of chronic pain on individuals’ lives, it remains crucial to emphasize the importance of using assessment tools that capture its various components during the treatment process. The results highlight the significance of both medical treatment and psychological factors, such as pain self-efficacy and partner’s emotional support, in influencing the manifestation of RA pain | | |  |  |
| Chung 2016,  Asia | | Cohort study | The psychosocial characteristics of family members in comparison with the general population. | | | | 367 RA family members51.7 years +/-18.8 SD  1101 matched controls 51.8 years +/-18.9SD  40% females in both groups | | Stress  Depression | | Family within the’ home above 19 years of age | | Stress and depression were more common in family members of RA people. | | |  |  |
| Coty 2010,  North America | | Cross sectional study | The relationship of problematic social support and family functioning to measures of subjective well-being in women with RA. | | | | 73 people with RA  29-92 years with mean age 57 years  100% female | | Negative social support  Unavailability of emotional support  Family functioning  Subjective well-being (Satisfaction with Life, Negative Affect and Depression)  Satisfaction with Life Scale | | Family within the household | | The degree of subjective well-being was jointly related to perceptions  of problematic social support and how well their families functioned, even controlling for demographic and health-related confounding variables. | | |  |  |
| Galloway 2021,  Europa | | Cohort study | The burden of disease from the perspectives of the patient and  caregiver | | | | 102 people with RA and 38 caregivers  People mean 55,5 years +/-13.9 SD  Caregivers mean 65.1 years +/- 12.7 SD  Patient 78% female  Caregiver 35% female | | Quality of Life  Disability  Pain  Fatigue  Morning Joint Stiffness  Work Productivity and Activity Impairment | | Caregiver | | Moderate RA requires frequent hospital visits for clinic appointments and has a considerable impact on the lives of people (and their caregivers) who are not eligible for advanced therapies based on current access restrictions. | | |  |  |
| Holtzman 2007,  North America | | Cohort study | Test whether satisfaction with responses would attenuate the detrimental lagged effects of morning catastrophizing on evening pain and negative affect. | | | | 62 people with RA  29-82 years mean 59 years +/- 10,9 SD  84% female | | Pain intensity  Pain catastrophizing.  Negative affect.  Satisfaction with spouse responses. | | Partner | | Day-to-day satisfaction with spouse responses can help reduce catastrophizing and protect against its detrimental effects. | | |  |  |
| Kasle 2008,  North America | | Cross sectional study | Psychological and physical health outcomes in relation to couples perceptions of and potential sex differences. | | | | 148 people with RA  Mean 56,6 years +/-12,3 SD  77% female | | Positive affect  Overall mutuality  Depressive symptoms  Anxiety symptoms  Physical disability  Arthritis impact | | Wife; Husband; Partner | | Mutuality, measured as perceptions of responsiveness in couples’ communications, is linked with better physical and psychological health in both men and women with RA. | | |  |  |
| Laidmäe 2009  Europe | | Cross sectional study | The problems aggravating the quality of life of people with rheumatism and outline the role of support received from their family in order to cope. | | | | 808 people with RA  66% was older than 56  85% female | | Hardships in daily life  Stress level  Assistance from the family | | Family | | Severe problems and stress symptoms display a cumulative effect – the occurrence of one aspect often triggers others. To break this vicious circle, people with rheumatism need assistance and support from the state as well as from their immediate environment | | |  |  |
| Lam 2009,  North America | | Cross sectional study | Examine the prospective relationship between spouse depression and RA disease course. | | | | 133 RA couples  People 29-86 years mean 62,35 years +/-12.65 SD  Spouses 28-86 years mean 63,26 years +/-12.79 SD  People 73% female  Spouses 29% female | | RA disease activity  Symptoms of RA (pain, swelling, tenderness, stiffness, and fatigue)  Physical limitations.  Disabilities of the Arm, Shoulder, and Hand  Depressive symptoms | | Wife; Husband; Partner | | Our findings highlight the key role played by the spouse in people with RA disease course and point to the importance of including the spouse in clinical interventions. | | |  |  |
| Li 2021,  Asia | | Randomized controlled trial | To explore whether family caregiver nursing education (FCNE) works on people with RA  Intervention consisted of 6 months of family caregiver nursing education. | | | | 158 RA pairs  People mean 59,2 years +/- 10,9 SD  Caregivers mean 47,4 years +/- 8,4 SD People 84 % female  Caregiver 72% female | | Outcomes:  Disease activity  C-reactive protein  Erythrocyte sedimentation rate  Tumor necrosis factor  Tender joint counts  Swollen joint counts Pain  Patient global  Health assessment Depression Anxiety | | Wife; Husband; Partner; Adult Child; | | The effect of FCNE on RA is multifaceted, weakening inflammation level, alleviating disease activity and relieving mood disorder. Relationship between caregiver and patient, caregiver’s education level and patient’s age may act as impact factors of FCNE. | | |  |  |
| Morgan 2015,  Europe | | Cohort study | The level of adherence and determine the relative contribution of demographic factors, RA disease-specific influences and psychological behavioral  influences on adherence. | | | | 329 people with RA  mean 55.92 years +/-12.27 SD  78% female | | Adherence | | Family | | In conclusion, a quarter of people showed only low to moderate adherence. Medication beliefs were associated with lower adherence. Increased professional/family support, stronger perceived illness chronicity and an increased feeling of treatment control also predicted adherence over time. | | |  |  |
| Pitsilka 2015,  Europe | | Cross sectional study | Investigate the association of structural and functional facets of social support with quality of life. | | | | 127 people with RA  33-79 years mean 60,7 years  84% female | | Quality of Life questionnaire  Patient Activity  Quality of Social Support  Social Network  Reciprocity | | Social support* | | Social support was a significant predic­tor of QoL, even when disease activity, demographic factors and social inte­gration were taken into account. Struc­tural aspects of social support were not significant predictors of QoL. | | |  |  |
| Pow 2018  Europe | | Cohort study | Examine the roles of both partners’  perceptions of esteem/emotional, solicitous, and negative support  in predicting subsequent shifts in pain. | | | | 27 RA couples  Patient 42-82 years mean 61.1 years +/- 10,5 SD Spouses 46-85 years mean 62.9 years +/-9,1 SD  People 72% female  Spouses 34% female | | Pain  Spouse Support Mobilization | | Partner | | Spouse responses play a key role in promoting adaptation in individuals coping with chronic pain. Within the limitations of the current study and sample, our findings advocate for the expression of love and acceptance to individuals with chronic pain. They also advocate against expressions from the provider of worry about or criticism of the recipient. | | |  |  |
| Rat 2021,  Europe | | Cross sectional study | Analyze the impact of the spouse’s health state and the patient–spouse relationship on functional impairment, mental health and self-efficacy of the patient with IA. | | | | 88 SpA, RA couples  People mean 59 years +/- 12,6 SD  Spouses mean 60 years +/-13,5 SD  People 77% female  Spouses 27% female | | Quality of life  Self-efficacy  Intimacy in relationships Dyadic adjustment  Social support Perceived burden  Comorbidity Mental health | | Husband; Partner | | Couple communication (experiencing an open and fluent exchange of ideas) in the patient’s mental health and self-efficacy but also perceived satisfaction with social support of both members of the couple on the patient’s mental health is important. | | |  |  |
| Ru 2019,  Asia | | Cross sectional study | Determine the degree of burden and depression on caregivers of people with RA and identify the characteristics of both groups that contributed to that distress. | | | | 195 people with RA  Mean 47,63 years +/- 15.23 SD  72% female  195 caregivers  Mean 47,87 years +/- 14.22 SD  45% female | | Care Burden  Depression  Disability  Health related quality of life | | Wife; Husband; Adult Child; Parent; other not described | | Caregivers of people with RA carry a moderate burden and have a low overall occurrence of depression, although one‐third of caregivers are still at risk for depression. Caregivers with poor health, less education and a closer relationship with the patient bear a relatively heavier burden. | | |  |  |
| Sabaz Karakeci 2018,  Asia/  Europe | | Cross sectional study | Association between commitments and responsibilities of the family caregiver of RA people  to the disease activity. | | | | 240 (60 people with RA and their 60 caregivers and 60 OA people with their respective 60 caregivers)  RA people mean 50,4 years +/- 11,1 SD  RA caregivers 43.1 years +/-15.3 SD  RA people 87% female  RA caregivers not described | | Disease severity  Pain  Disease activity  Caregiver reaction  Caregiver strain  Tasks related to care, household, and assistance and allocated time periods for each group | | Caregivers | | Disease activity has a considerable impact on the commitments and responsibilities of individuals who primarily provide care for RA people. | | |  |  |
| Strating 2007  Europe | | Cross sectional study | Understand which primary and secondary stressors determine patient’s distress and which determine partner’s distress. | | | | 61 couples  People with RA mean 60.1 years +/- 11.5 SD  67% female  Partners mean 59.8 years +/- 13.8 SD  33% female | | Distress  Functional disability  Partner burden  Negative transactions  Perceived marital quality | | Wife; Husband; Partner | | Disability was found to be a primary stressor of their distress. In addition, it also had an indirect effect on partners’ distress through partners’ primary stressor, perceived burden. Marital quality and negative transactions can be considered joint secondary stressors for partners, not for people. | | |  |  |
| Uludag 2012,  Asia/  Europe | | Cross sectional study | Investigated the quality of life  and the rates of depression in spouses/partners of people with AS compared with healthy controls. | | | | 25 AS spouses and 25 control spouses  Spouses mean 35 years +/- 6.47 SD  Control Spouses mean 36.26 years +/-5.93 SD  84% female in both groups | | Depression | | Wife; Husband | | Being a spouse of a patient with AS significantly interferes with quality of life and increases the depression frequency as much as patient with AS. Spouses of AS people might also be investigated for depression and quality of life, and therapy program should be begun as soon as possible. | | |  |  |
| Yasuoka 2022 Asia | | Cross sectional study | Examine the psychosocial characteristics of patients with RA by remission status and to determine the impacts of instrumental and emotional support on severity of depressive symptom. | | | | 360 RA people  Mean age 64.9 +/- 9.8 SD  84.2% female | | Disease activity  Emotional support  Instrumental support  Physical function | | Social Support | | In conclusion, favourable association between emotional support and severity of depressive symptoms is confirmed only among RA patients in remission status. The influence of emotional support in non-remission patients and that of instrumental support regardless of remission status are inconclusive. The type of social support that RA patients need may vary by remission status. RA patients with good control of disease activity may need to be cared for depressive symptoms when they are having no emotional support | | |  |  |
| Öksüz 2021,  Asia/  Europe | | Cross sectional study | Evaluate the effects of loneliness, perceived social support, and depression on the medication adherence of people with AS. | | | | 119 AS people  mean age 35,80 years +/- 9.08 SD  12% female | | Disease status  Medication adherence  Social support  Depression | | Family | | Age, loneliness, perceived social support, and depression are important factors related to medication adherence of people with AS. Nonadherent people with AS had lower perceived social support and higher levels of loneliness and depressive symptoms. | | |  |  |
|  | |  |  | | | |  | |  | |  | |  | | |  |  |
| Study ID Continent | | Aim | Participant characteristics | | | Setting | | Themes | |  | Type of significant others | | Author conclusion | | |  |  |
| **Studies applying a qualitative design presented in alphabetical order** | | | | | | | | | | | | | | |  |  |  |
| Alfaro 2013  Europe | | Perceptions, attitudes, and experiences of relatives. | | 18 informal AS, RA caregivers  31-65 years  61% female | | Three Rheumatology departments and three patient associations.  Two discussion groups, 90 minutes duration. | | - Alterations in daily life - Caregiver support - Impact on the caregiver - Patient characteristics - Description of care | | | | Informal caregiver | Caregivers experience loss of purchasing power, labour problems, social isolation, and emotional overload. | |  |  |  |
| Backman 2007  North America | Impact of IA on the role of mother? | | | 12 People three with AS, six’s with RA, one JIA, two with SLE.  24-53 years  100% female | | Through a university-affiliated arthritis program. Individual semistructured in-depth interviews 60-120 minutes. | | - Participation in Mothering Tasks - Kinds & Level of Support - Balancing Energy & Fatigue - Impact on the Family | | | | Partner; Mother; Grandparent | Inflammatory arthritis has a dramatic impact on the experience of motherhood, with both positive and negative influences.. | |  |  |  |
| Bergstrøm (Nov) 2021 Europe | How id support expressed in the dyadic relationships and how support can influence  participation in everyday life of persons with RA. | | | 16 RA (dyads)  People 34-70 years with mean age 62 years  Significant other not stated.  People 50% female  Significant others 69% female | | Participants were recruited from the TIRA cohort. Individual semistructured interviews 18-71 minutes. Dyads interviewed separately. | | - Time is a friend - The dynamic of support - We need support from others - What we share with each other | | | | Wife; Husband; Partner; Friend; Daughter; Mother | Different aspect and dynamics of support occur in everyday life of persons with RA and their significant others. Both parties expressed that this reciprocal support had become a natural part of everyday life over time, especially emotional support. They also described that well-functioning communication facilitated participation in everyday life, and that people outside of the dyads were important sources of support. | |  |  |  |
| Bergstrøm 2020 Europe | The meaning of significant others in relation to participation in everyday life of persons with early diagnosed and contemporary treated RA. | | | 59 people with RA  22-64 years  58% female | | Participants were recruited from the TIRA cohort. Individual semi structured interviews 45-90 minutes. | | - My early RA causes activity adaptions for us all - Making the significant others balance between shortfalls and participation*)* - Physical interactions with significant others - Emotions in relation to activities with significant others*)* | | | | Wife; Husband; Partner | Significant others can facilitate as well as hinder participation in everyday life for persons already during the first years after diagnosis of RA, through their attitudes and actions. Adapting activities with a positive attitude can be a way to facilitate participation, whereas misunderstanding symptoms or pointing out a need for help can hinder participation. | |  |  |  |
| Brignon 2020  Europe | Explore people’ and relatives ‘experience of IA and their difficulties, relationship, communication,  coping strategies and needs. | | | 20 patient dyad seven with SpA, 13 with RA.  Patient 27-79 years mean 63,0 years  40% female  Significant other 39-83 years mean age 60 years  65% female | | Participants were recruited from seven rheumatology departments. Individual semistructered interviews 60-90 minutes. Dyad was interviewed together. | | - Disease lived together - Impact of the disease on the relationship - Difficulties and needs of the relative - Social impact of the disease on the dyad and shared difficulties | | | | Spouse; Daughter; Friend | This study highlighted the importance of recognizing the role of the relative in the management of IA disease, especially when medical decisions are shared with professionals. A joint approach to treatment is a basis for coping with the disease. | |  |  |  |
| Brodin 2023  Sweden | To qualitatively explore and describe the management of pain among patients with PsA | | | 11 PsA people  Mean age 52 (40-67 range)  73% female | | Individual face-to-face or telephone interview 24-59 minutes. | | - Sorting out vulnarbility - Reaching acceptance and engagement - Directing focus to change | | | | Family | Managing the constant murmur of pain in PsA by taking charge of life includes sorting out vulnerability, reaching acceptance and engagement, as well as directing focus toward change. Understanding the action components of pain management from a theoretical perspective highlights the importance for patients of satisfying three basic psychological needs: competence, autonomy, and relatedness. | |  |  |  |
| Chilton 2021  Europe | Explore Primary Care factors in relation to early referral decisions to Secondary Care. | | | 11 people three with early IA and eight with early RA.  People  44-72 years  64% female | | Participants were recruited from 2 rheumatology outpatient departments. Individual in depth semi structured interviews up to 60 minutes. | | Navigational Struggles   - - Persuasion of family   - Lack of continuity in care   - Pushing for referral   - Strained relations   - Lost time | | | | Wife; Husband; Partner; Parent; Friend | Participants reported that they didn’t feel listened to, believed or understood and they associated this with their delays to referral into Secondary Care. The consequences of this delay extended from people to their families, as people relied on support from their family members to pursue their referral. Participants reflected on this as a period of great frustration and  perceived that this impacted on their relationship with their GP | |  |  |  |
| Dures 2017  Europe | People’ views and experiences on psychological support for their IA. | | | 779 people with IA.  mean 59 +/-12,6 SD  80% female | | Open ended questionnaire distributed to people at 6 rheumatology units and a national patient RA charity. 64% response rate. | | - - Challenges of altered life course   - Understood by *others*   - Acquiring strategies | | | | Wife; Husband; Partner | Reports of high levels of psychological distress were commonplace among people with IA and often attributed to the impact of pain and fatigue. The consequences included negative emotional responses, including depression, and withdrawal from social interactions, leading to loneliness and isolation. In addition to valuing the support of family and friends, people looked to the rheumatology team to acknowledge the psychological impact of their IA. Although this was often not provided, when it was offered, people identified clinicians’ understanding and signposting to appropriate support as helpful. | |  |  |  |
| Fallatah 2015  North America | What are the experiences of family members and what forms of social support are needed by family members to provide  adequate support to their relative with RA. | | | 7 RA family members  People 45-79 years  Family member 25-74 years  Patient 86% female  Family member 42% female | | The Arthritis Society and two primary health care clinics, word-of-mouth, KijijiÂ© and CraigslistÂ© Individual interviews 60-90 minutes. | | - - Effect of the disease   - Reshaping the relationship   - Provider of support   - Social support needs of family members   - Finding balance and coping | | | | Wife; Husband; Adult; Child | The social support needs of family members are influenced by their individual situation and social relationships with their relative with RA and others in the social network. | |  |  |  |
| Jahani 2022  Asia | Explain the lived experiences of people with RA | | | 24 people with RA  21-60 years  67% female | | Participants were recruited from one rheumatology clinic and one rheumatology ward. Individual unstructured interviews 20-65 minutes. | | - Self-management with the family’s participation - Spirituel resilience in the face of problems - Tendency to hide the disease - The fear of an uncertain future | | | | Family | The findings of the present study show that people with RA, despite various physical, psychological, medication, and social problems, can strengthen their self‑management with family support and try to cope with their fears and worries by resorting to spiritual issues. | |  |  |  |
| Kostova 2014  Europe | Identify the key  sources and types of social support that are relevant for rheumatoid arthritis  people | | | 20 people with RA  35-69 years  65% female | | The Swiss Association of Rheumatology and 3 rheumatologists. Individual interviews 60-90 minutes. | | - Family (Facilitators, Barriers) - Physicians (Facilitators, Barriers) - The external social context: significant others (significant others) | | | | Wife; Husband; Partner | We conclude that sources of social support need to find a middle way between skepticism and solicitousness. | |  |  |  |
| Matheson 2010 Europe | Insight into the experiences of the partners of people with RA. | | | 8 RA partners  48-73 years  25% female | | Participants were recruited through a rheumatology clinic. Semi-structured interviews 40-80 minutes. | | - Psychological burden - It’s a restricted life - Adjusting lives - It’s a joint approach - Met and unmet support needs | | | | Partner | In conclusion, partners of people with RA reported supportive strategies that were vital to the people’ disease management, but the data show that many carry a substantial psychosocial burden. | |  |  |  |
| Parton 2022  Australia | How do mothers with RA subjectively experience wellbeing and coping? | | | 20 people with RA  23-54 years mean 36.6 years  100% female | | Through an online survey advertised through social media. In depth induvial telephone interviews 60 minutes. | | - Burden and complexity in the mothering role - Losing control: Woman’s experiences of psychological distress | | | | Grandparent; Uncle; Aunt; Husband | The findings from this study suggest that mothers who live with rheumatoid arthritis maternal subjectivity, with their own RA management concerns, highlighting the complex negotiation of coping and wellbeing for women living with RA experience additional burden and complexity in their role. Women can be seen to manage both risk to a ‘good’ | |  |  |  |
| Rai 2018 North America | Explore IA people’ perspectives on tools and strategies to support chronic medication use. | | | 27 AS (5), + more RA (15), PsA (5) SLE (1), IA (1) people  20-79 years  63% female | | Participants were recruited from rheumatology clinics. Focus group interviews 120 minutes. | | - Adapting to life with inflammatory arthritis - Complexities and dynamic nature of taking medications - Developing lifestyle strategies for medication use - Becoming informed about medications - Receiving support | | | | Wife; Husband; Adult child | Beyond confirming the roles of information and support as facilitators of medi­cation use, we have established a framework that identifies practical and actionable targets for patient-oriented adherence interventions for IA. | |  |  |  |
| Raybone 2019  Europe | Explore how and  why axSpA impacts on partner relationships according to  individuals with axSpA and their partners. | | | 9 SpA couples  23-65 years for both groups  People 67% female  Partners 33% female | | Participants were recruited via social media pages (Facebook and Twitter). In-depth semistructured telephone interviews with both members of the couple separate 25-73 minutes. | | - Perceived relational closeness - Playing third wheel to axSpA - Tension surrounding a carer-type role | | | | Partners | All dyads experienced difficulty with managing axSpA, from activity restrictions to changes in relational strength and altered relational roles. Specifically, findings identified a need to provide support which meets the needs of both individuals with axSpA and their partners. Information should be provided to educate and prepare partner dyads about axSpA’s potential impact on relationships and effective methods of dyadic coping and thought given to more extensively including partners in treatment where appropriate. | |  |  |  |
| Stamm 2010  Europe | Explore how contextual factors affect the everyday activities of women and men with rheumatoid arthritis (RA), | | | 15 RA people  Females 42-63 years mean 52.4 years +/-8.7 SD  Males 38-58 years mean 51.8 +/-9.3 SD  73% females | | Qualitative analysis of a narrative biographical study from 2008. individual interviews were conducted with each participant 60-120 minutes. | | - Life stories of women - Life stories of men | | | | Family | Interventions by health professionals in people with RA may benefit from an approach sensitive to personal and environmental factors | |  |  |  |
| Stoll 2024  Europe | Provide evidence on how to combine health and social resources with ‘traditional treatments’ (ie, drug therapy) to improve care and inform resource allocation and service redesign, ensuring fair and equal access to all | | | 45 RA people  Age 25 and above  84% female | | Thematic analysis of online individuelle interview and focus groups 30-90 minutes | | - Life ecperiences and wellbeing practices - Patient education and health literay - Socioeconomic (dis)advantages - Employment and working conditions - Social and familial support - Helathcare services | | | | Family | In conclusion, this study adds to the evidence that beyond biological factors, psychosocial factors also play a role in health and RA management50 51 and highlights the value and need for clinicians to ask, support, signpost and record psychosocial contexts, and holistic care and treatment. Taking a collaborative and integrated biopsychosocial care approach can enhance patient care and experience, minimising the risk of treatment failure and maximising health potential for all, irrespective of social background. | |  |  |  |
| Sumpton 2022 Australia | Describe people’ experience and values regarding shared decision-making to inform ways to improve  patient-centered care in people with PsA. | | | 25 PsA people  27-79 years  44% female | | Participants was recruited from 2 tertiary referral centers. Individual semistructured interviews 27-58 minutes. | | - Lacking agency in decision-making - Overwhelmed by potential harms - Gaining confidence - Opting for alternatives - Building trust and fortifying collaboration | | | | Family | A lack of knowledge, lack of provision of treatment options, a perception of automated treatment algorithms, and the necessity of treatment disempower people with PsA from being active members in shared decision-making about medication taking. This lack of agency is compounded by having to contend with multiple potential harms of systemic treatment. People with PsA gain confidence in decision-making with time and rely on consistent and open communication from health professionals to build trust in being active members in these decisions. | |  |  |  |
| Tiwana 2015  Europe | Explore the influence of family and friends in help-seeking  decisions at the onset of RA and how help-seeking behaviour was influenced by significant others. | | | 11 RA people and 8 significant others  Overall, 18 to 90 years mean 54 years  Overall, 68% female | | A clinic database. Individual semistructured interviews was conducted separately. Length of interview not stated. | | - The impact of early symptoms on significant others - Communicating to significant others that there was a problem - Significant others’ belief about the possible causes of symptoms - The influence of significant others in encouraging prompt help-seeking | | | | Wife; Husband; Adult child; Friend | Significant others play an important role in influencing help-seeking behaviour; this has implications for theoretical models of help-seeking and the development of help-seeking interventions. A negative consequence of social interactions resulted from a lack of understanding and knowledge about RA among significant others, highlighting the need for greater public awareness about the early symptoms of RA. | |  |  |  |
| Untas 2020  Europe | Explore people’ and relatives’ perceptions  and experiences about the role of relatives in disease  management. | | | 20 SpA couples  People mean 59,5 years  Relatives mean 60 years  People 70% female  Relatives 40% female | | In 7 departments. Semistructured interviews with the patient and relative together was conducted 60-90 minutes. | | - Perception of relatives’ general roles expected regarding the disease - Dyad relationship - Help relationship | | | | Wife; Husband; Partner | The study shows that people and relatives have similar perceptions regarding the relatives’ role toward people with IA: knowledge about the disease, technical skills and interpersonal skills. This refers to self-care and psychosocial skills in therapeutic education programs. | |  |  |  |
| Study ID Continent | Aim | | | Participant characteristics | | Setting | | Themes | | | | Type of significant others | Author conclusion | | Type of significant others | | Author conclusion |
| **Study applying a mixed method design** | | | | | | | | | | | | | |  |  |  |  |
| Pile 2020  Asia & Australia | | This study investigated the roles and potential impact of carers in RA management in the Asia-Pacific region | | | Qualitative part:  10 rheumatologist, 10 people,  10 caregivers  Age and sex not available  Quantitative part: 131 rheumatologist, 382 people,  395 caregivers  Australian people mean 54.6 years +/- 15.3 SD 63% female  Chinese people mean 43.6 years +/- 11.5 SD 56% female  Japanese people mean 55.9 years +/- 12.6 SD 55% female  Australian carers 48% female  Chinese carers 65% female  Japanese carers 29% female | Qualitative part:  Participants was recruited in triads (treating rheumatologists, people living with RA, and their carers) at rheumatology departments across 3 countries.  Quantitative part: Online survey consisted of 3 separate questionnaires designed specifically for carers, people and rheumatologists. . | | Qualitative part:   - Role of the carer - Carer involvement in consultations - Carer support   Quantitative part:   - Role of the carer - Carer influence on treatment decision-making - Impact of carer observations - Support for carers | | | | Caregiver | Carers play an important role in RA management by providing physical, emotional, and financial support to people, especially for people with moderate-to-severe disease. They may also help to optimize treatment outcomes by reinforcing important information about the disease and treatment and providing observations that may help rheumatologists in treatment decision-making.  While stakeholders considered carer observations to be valuable, they were obtained on an ad hoc basis. Any impact on clinician decision-making by carer-reported measures will also require investigation and careful assessment of acceptability to all stakeholders in different settings. Finally, integration of carer-reported outcomes into clinical discussions should not interfere with workflow or add to the workload of rheumatologists. |  |  |  |  |
